# Proposing a Conceptual Framework: Social Media Infodemic Listening for Public Health Behaviors

**DOI:** 10.3389/ijph.2024.1607394

**Published:** 2024-11-14

**Authors:** Shu-Feng Tsao, Helen Hong Chen, Samantha B. Meyer, Zahid A. Butt

**Affiliations:** School of Public Health Sciences, Faculty of Health, University of Waterloo, Waterloo, ON, Canada

**Keywords:** health infodemic, social media, social listening, conceptual framework, machine learning

## Abstract

Various communication and behavioral theories have been adopted to address health infodemics. However, there is no framework specially designed for social listening studies using social media data, machine learning, and natural language processing techniques. We aimed to propose a novel yet theory-based conceptual framework for infodemic research. We collected theories and models used in COVID-19 related studies published in peer-reviewed journals, ranging from health behavior, communication, to infodemic studies. These were analyzed and critiqued for their components, and we subsequently proposed a conceptual framework with a demonstration. Accordingly, we proposed our “Social Media Listening for Public Health Behavior” conceptual framework by not only integrating important attributes of existing theories, but also adding new attributes. The proposed conceptual framework can be used to better understand public discourse on social media, and can be integrated with other data analyses to gather a more comprehensive picture.

## Introduction

The World Health Organization (WHO) has consistently reiterated the widespread and multifaced nature of health infodemics and their harmful consequences throughout a pandemic [1]. According to the WHO, health infodemics represent an excessive amount of information, both misleading and trustworthy, spread in digital and physical environments about an acute public health crisis during its outbreak [1]. The WHO has hosted infodemic conferences and training since early 2020 to address increasingly complex health infodemics because they have compromised public health measures by causing confusion, risky behavior, and decreased trust in health authorities [1]. The WHO’s technical consultation has led to a framework to manage infodemics [1], resulting in recommended strategies, practices, and toolkits of infodemic management from preventions to reactions for health professionals [1].

Another framework that categorizes research agendas for infodemic management was developed from the first WHO’s infodemic conference [1]. Before infodemics can be managed, it is necessary to measure and understand them. Over the course of the COVID-19 pandemic, systematic reviews showed that health infodemics, especially those with misleading health information, were prevalent and far-reaching on social media before and during the pandemic [2]. Depending on social media platforms, health misinformation can account for less than 1% to almost 30% of user-generated content [2]. Vaccine hesitancy fueled by health misinformation accounted for over 30% of the studies included in the systematic reviews [2]. However, various theories have been used to guide studies of health infodemics on social media [3]. Different theories have suggested inconclusive predictors, mediators, and moderators, but scholars have constantly regarded behavioral intentions or behavior as the ultimate outcome, yet the measurements thereof have varied [3, 4]. Additionally, further research is needed to understand how online infodemics have influenced offline behavioral intentions or behavior [4]. The WHO has repeatedly called for multidisciplinary collaborations since professionals in communications, neuroscience, and digital marketing have long studied how social media manipulates people’s behavior [1].

With the advancement in machine learning (ML) and natural language processing (NLP), infodemic research applying different ML or NLP techniques to analyze social media data to understand public discourse has grown exponentially. For example, the WHO has developed and deployed an “Early AI-supported Response with Social Listening” (EARS) platform to identify emerging information voids following the WHO’s terminologies [5]. Nonetheless, existing social listening tools, given their marketing-driven designs, need more customization to meet the needs of infodemic social listening platforms like the EARS [5]. In a public health crisis, health professionals need a tool that can efficiently harness and analyze tremendous amounts of online data to understand public discussions in a timely manner since qualitative analysis is time-consuming.

The latest ML and NLP techniques, including topic modeling, sentiment analysis, and stance detection, have been used in infodemic social listening [6]. For example, several sentiment analysis techniques, such as Bidirectional Long Short-Term Memory (Bi-LSTM) and Valence Aware Dictionary and sEntiment Reasoner (VADER), have been used to categorize emotions [6]. These techniques can classify eight different emotions, or simply classify emotions as positive, neutral, or negative [6]. Although improvements are still needed to decrease misclassifications in these supervised and unsupervised ML and NLP techniques, they have been widely applied during the pandemic for social listening studies [6]. These ML and NLP techniques are commonly used as a screening layer to quickly understand public discourse at a superficial level. Therefore, qualitative analyses can be conducted to further understand or identify information voids from the conversations.

Researchers have applied these advanced ML or NLP techniques to infodemic studies along with existing health theories, such as the Health Belief Model (HBM), social cognitive theory (SCT), and social-ecological model, and tools to overcome challenges in generating new tools given limited resources [1]. Although these health theories have been long established, most are developed before the existence of social media [3]. Ubiquitous social media has changed how people consume and behave regarding online health information, for better or for worse [4]. Schillinger et al. constructed a Social media and Public Health Epidemic and Response model [7]; and the Aral Hype Loop [4] demonstrates that social media has both perils and merits. That is, social media can help people make informed decisions, while also spreading harmful misleading information [6]. The WHO has recommended that social listening for infodemic management should be incorporated into future pandemic preparedness [1].

During the pandemic, social listening was mostly reactive rather than proactive. Health professionals and public health organizations rushed to debunk misinformation while competing for people’s attention to urge them to follow evidence-based preventive behaviors during times of uncertainty [1]. Although many lessons have been learned regarding health infodemics using existing theories and tools, there is a need to carry out social listening in a systematic way based on a novel theoretical framework for health researchers. Except Aral’s Hype Loop [4], which was developed entirely based on social media, other theories or frameworks were developed before the existence of social media. We, therefore, aimed to propose a conceptual framework that helps monitor public discourse on social media and behaviors for future infodemic research. The proposed framework aims to investigate how people’s emotions and attitudes are associated with their online behavior on social media, and their offline behavior in the real world, given health information pushed by social media algorithms.

## Methods

The theory construction methodology (TCM) by Borsboom et al. was adapted to develop a conceptual framework given its practical and flexible methodology [8]. According to TCM [8], there are five steps as follows: (a) identification of relevant phenomena; (b) development of a proto theory; (c) formation of a formal model; (d) adequacy evaluation of the formal model; and (e) assessment of overall worth of the formal model [8]. Firstly, we identified the phenomenon of interest as how online health information on social media can influence people’s behavioral intentions or behavior during the COVID-19 pandemic. Next, we conducted a theory synthesis [9] to develop a conceptual framework. We searched PubMed, Scopus, PsycINFO, and Google Scholar databases for theories used in reviews and original research manuscripts related to social media infodemic research. Keywords included “social media,” “online discussion,” “public discourse,” “behavior,” “intention,” “attitude,” “perception,” “theory,” “model,” “framework,” and related synonyms, but explicitly excluded “conspiracy theory” in the search. We included articles written in English published in peer-reviewed journals from December 2020 to December 2022.

Reviews were prioritized for extraction and reading if they summarized common theories used in COVID-19 related social media infodemic studies. The search for relevant theories in this process was non-exhaustive. A total of 13 theories were included for Walker and Avant’s theory synthesis [9]. Constructs of the included theories were individually evaluated by S-FT, HC, and ZAB with regards to our phenomenon of interest to identify common components and how social media infodemic studies have categorized them using ML or NLP techniques following a qualitative codebook developed by S-FT. The codebook was included in Supplementary Table S1 with expertise from SBM for qualitative coding, as well as HC and ZAB for their expertise in ML or NLP techniques. After theory synthesis, a formal conceptual framework was proposed with explanatory descriptions.

## Results

### Synthesis of Theories

Supplementary Table S2 shows the thirteen theories included in this study. As expected, the HBM is commonly used; one systematic review reported that HBM was used in 126 social media infodemic studies regarding COVID-19 vaccine hesitancy [10]. Some existing theories are also combined or adopted by researchers to investigate complex and multifaceted health infodemics in various social media studies. For example, the theory of planned behavior (TPB) is an extension of the theory of reasoned action (TRA) [10]. TPB is combined with the heuristic systematic model to create the risk information seeking and processing model (RISP) [11] or integrated with the uses and gratifications theory to investigate information-sharing behaviors [12]. Furthermore, Scannell et al. [13] weaved the social judgment theory, elaboration likelihood model of persuasion (ELM), and extended parallel process model (EPPM) to understand how persuasive COVID-19 vaccine (mis)information was to convince people, implicitly affecting their behaviors [13]. Overall, these studies demonstrated various theoretical approaches used to investigate how social media health infodemics have impacted people’s intentions or behaviors.

Throughout these theories, several factors have been shown to influence behavioral intentions or behaviors. Although they are described in different terms, they are used interchangeably in most contexts. For instance, “self-efficacy” in HBM and SCT has shared a similar meaning with “confidence” in the behavioral and social drivers (BeSD) of vaccination, “perceived behavioral control” in TPB, and “efficacy” in EPPM. If the meaning is extended further, it can also represent “capability” in the model of capability, opportunity, and motivation leading to behavior (COM-B), “ability” in ELM, “behavioral capability” in SCT, “Act” in the Hyper Loop, and “behavioral intention” in TPB/TRA, and the Transtheoretical Model. Another group of terms—attitude, perceptions, and motivation—can also share comparable meanings. Five of the thirteen theories include “attitude,” another three theories consist of “motivation,” and the other two theories involve perceived variables that are associated with the outcome. These components have suggested that people’s views are consistent or in contrast with given health information on social media. In addition, these components have suggested gaps between “self-efficacy” and “(cap)ability,” “perception” and “reality,” or “subjectivity” and “objectivity.” However, it can be challenging to distinguish these components because they shape each other, or it is too difficult to measure them separately. Similarly, attitudes and perceptions may be indistinguishable as they both imply motivations or intentions for behavioral uptake or changes.

Almost all theories focus on individual behaviors. However, several theories, including HBM, SCT, and BeSD, have incorporated variables beyond personal levels to infer behavioral intentions or behaviors [10–13]. Unlike EPPM, these models do not explicitly measure emotional variables, although they might be inferred in variables related to self-efficacy, perceptions, or subjective norms. One implicit assumption in these theories is that people can determine and behave rationally to mitigate risks if they perceive greater threats or susceptibility to themselves. Nonetheless, the latest social media infodemic research has shown otherwise [4, 5]. That is, behaviors may not be completely driven by rational reasoning [3, 4]. Prior studies have shown how social media, given artificial algorithm designs, can manipulate or help spread emotional posts, making them spread far and wide [4]. Therefore, emotion should also be considered when inferring behaviors, similar to perception, attitude, motivations, and others.

Given limitations and gaps identified in existing theories and frameworks, a new conceptual framework is needed to reflect the current complex health infodemic issues in today’s information ecosystem, especially on social media [1–3]. The new conceptual framework should incorporate theories from various fields. In addition, components measuring attitudes and emotions should also be included in the framework.

### Proposed Conceptual Framework

**FIGURE 1:**
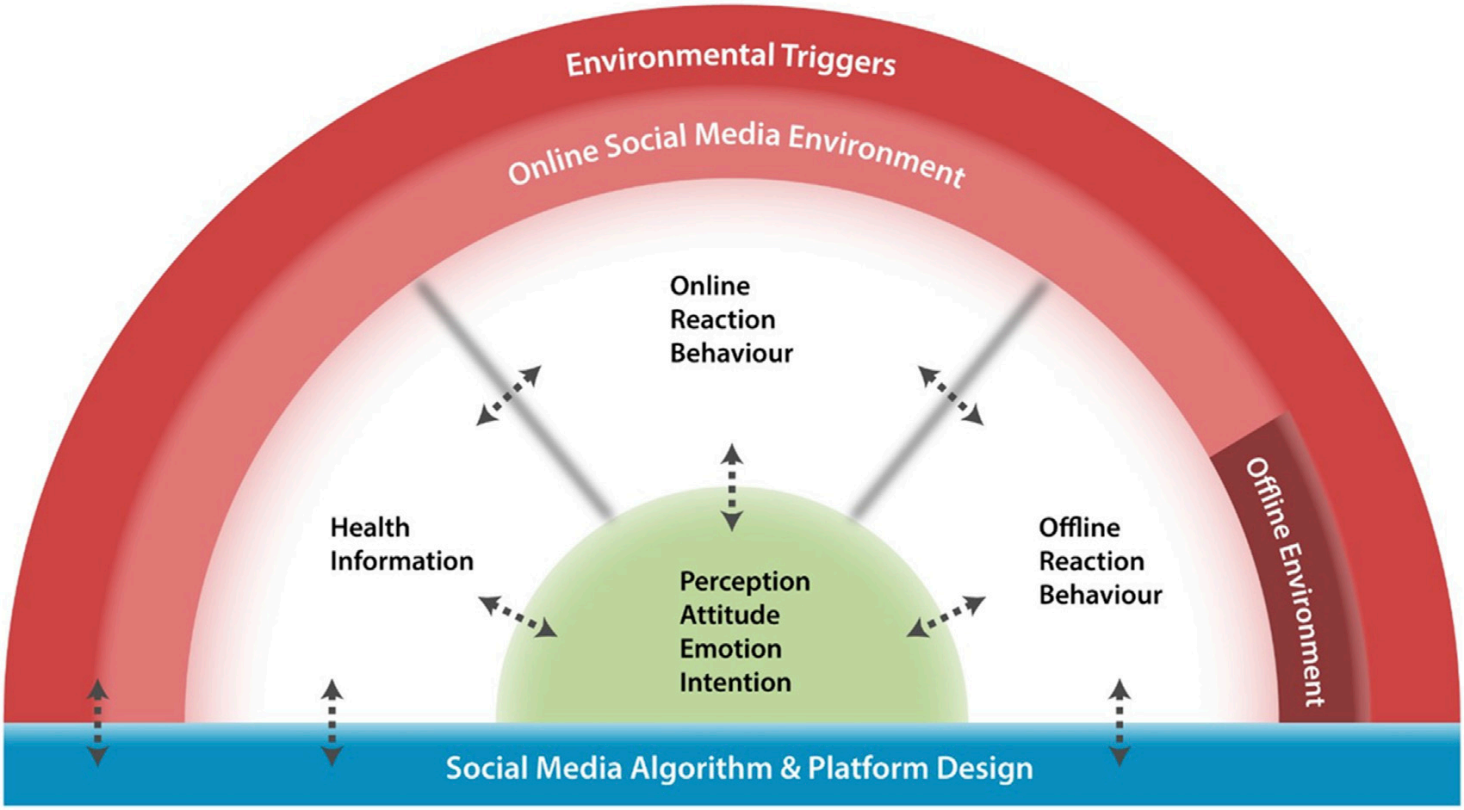
The proposed conceptual framework: Social Media Infodemic Listening for public health behavior. This framework addresses the identified gaps, including implicit emotional measures, implicit assumptions of rational behaviors, and active spread of health information driven by social media’s designs and algorithms. Double arrows illustrate potential associations between the five constructs. Blurry boundaries and faded colors demonstrate that the components can happen both online and offline simultaneously. (Proposing a Conceptual Framework: Social Media Infodemic Listening for Public Health Behaviors. Canada, 2024).

We propose a novel conceptual framework—Social Media Infodemic Listening (SoMeIL) for public health behavior (Figure 1)—to address the identified gaps, including implicit emotional measures, implicit assumptions of rational behaviors, and active spread of health information driven by social media’s designs and algorithms. Unlike existing theories, our framework no longer assumes rational judgments and behaviors. In the following sections, we will introduce and explain each construct illustrated in our proposed conceptual framework, along with some limitations in social media data or ML and NLP techniques.

#### Social Media Algorithm and Platform Design

As demonstrated [4], the social media algorithm has user attributes inputted (Table 1), such as demographic and historical behavioral data. The social media algorithms then “recommend” posts or people for users to read or follow, respectively, based on similarities in demographics or interests instead of differences [4]. Social media algorithms are intentionally designed to be addictive and affective [4]. The issue is further compounded by highly personalized user experiences on social media given people’s digital footprints, encouraging echo chambers or polarization [4]. Coupled with its engagement design, such as “like” and “follow” buttons, social media have kept their users spending more time on the platform as “engagements” [4]. Such characteristics are defined as “user attributes on social media algorithms” in the proposed conceptual framework (Table 1).

**TABLE 1:** Attributes of each component in the SoMeIL conceptual framework (Proposing a Conceptual Framework: Social Media Infodemic Listening for Public Health Behaviors. Canada, 2024).

Components	Attributes
User attribute on social media	Age Sex Geolocation Income Education Occupation Party affiliations Region affiliations Following Followed
Inferred intention	Attitude • Acceptance • Non-commitment • Rejection Emotion • Positive • Negative • Neutral • Mixed Perception Ideology
Online reaction behavior	Share Like/dislike Comment Post Bookmark Nothing
Offline reaction behavior	Agreement Disagreement

#### Health Information

This component represents information related to health circulating from users on social media. After the health information is inputted, the social media algorithms selectively “recommend” or “push” the health information, misleading or scientific, to the users according to the users’ profiles and historical usage. Scientific health information competes with misleading information for social media users’ attention.

#### Online Reaction Behavior

Next, we define “online reaction behavior” as it occurs “after” a user views health information. Collective online behaviors can be measured via the numbers of likes, shares, and others (Table 1). We are especially interested in online behavior, or its propagation patterns, because this can be used to infer or confirm collective intentions, as measured by the number of shares or likes. For instance, digital marketing research on Twitter has long estimated the number of users sharing similar opinions by the number of likes and retweets of a given tweet, whereas disagreements can be reflected by the number of replies since there are no downvote or dislike option. Therefore, by collecting and analyzing the attributes within the online behaviors, scholars can better understand or estimate what inferred intentions of the “quiet majority” users are since approximately 10% of users produce 90% of content on Twitter, for example [4]. Online behavior can be used to infer people’s behavioral intentions.

#### Perception, Attitude, Emotion, Intention

Existing models have theorized that behaviors can be attributed to attitudes, perceptions, and emotions, but it has remained challenging to clearly distinguish them because they are interrelated and cannot be easily measured [2]. Researchers have inferred associations among attitudes, perceptions, and emotions in various ways [6], but we decided to group these together in our framework as “inferred intention” (Table 1). In our opinion, it may be unnecessary to distinguish them since they can be used interchangeably or along with each other in different contexts. What really matters is to infer potential behavioral intentions using attitudes, perceptions, emotions, or any combination thereof. We adopted social judgment theory (SJT) to infer intentions (Table 1) because this makes it more feasible when using ML or NLP techniques to analyze social media data, especially in infodemic studies.

#### Offline Reaction Behavior

Although boundaries between our physical and digital worlds have become less distinguishable, it remains unclear whether people really react upon information received from social media. Some may have consistent online and offline reaction behaviors, contradictory online and offline reaction behaviors, and either online or offline reaction behaviors. Even if individuals tweet or like a tweet indicating that they are willing to get vaccinated, it remains inconclusive unless they later share a selfie or their vaccination record on social media to prove that they, in fact, received a vaccination. In this case, their self-reported offline reaction behavior matches their online reaction behavior. Their self-reported offline reaction behavior is also adherent to public health interventions. Therefore, one’s self-reported offline reaction behavior can be inferred in two ways: one is whether an individual’s online and offline self-reported behaviors are consistent, and the other is whether their self-reported offline behavior follows the public health intervention. The “offline reaction behavior” in the COVID-19 vaccination example has been primarily self-reported if using only social media data. However, there are other data, such as administrative data, which can provide directly measured “offline reaction behavior” instead of self-reported data from social media or survey.

## Discussion

The SoMeIL conceptual framework consists of five major constructs inspired from existing theories. Dashed boundaries indicate that online and offline environments have become less distinctive as information flows. Arrows represent potential associations among these components and how they influence or self-feed each other as the framework gives a sense of loop. Attributes of each construct can be inferred or measured via advanced NLP or ML techniques if data are available and of high quality. Although we have used ML or NLP techniques to explain our conceptual framework throughout this paper based on our study published during the COVID-19 pandemic [6], the proposed framework is not limited to quantitative infodemic research only. That is, the proposed conceptual framework can be applied in qualitative research.

There are several limitations in the SoMeIL conceptual framework. Firstly, since user attributes are voluntarily inputted by users when they create their social media accounts, most user attributes (Table 1) are optional, and values can be fictitious. In other words, they can have missing data, or even be false when values are not missing, although correct values exist. Some social media platforms require users to enter their email and password to create an account with a username without any other details. Therefore, the users can remain primarily anonymous or unverified on the platform. Geolocation is another special issue for researchers when modeling disease outbreaks or heat maps using Twitter data [14]. For example, tweets tagged with explicit geolocations can vary from less than 1% to approximately 4% of data collected from Twitter [14], depending on data collection methods and the amount of data collected. Although there are many ML or NLP techniques to infer geolocations for Twitter data [14], they are not as precise or comparable as internet protocol addresses. Furthermore, public discussions related to vaccinations on social media have become more polarized over time [14]. Studies have demonstrated that user attributes, such as political party affiliations, religious affiliations, and who to follow can potentially indicate ideologies or attitudes toward vaccinations [14]. Similar to the geolocation issue, researchers may not have direct access to these attributes. If users enter some information within these attributes, the accuracy thereof remains uncertain. Additionally, even if researchers apply advanced ML to infer these attributes, these techniques may be unable to generalize to other studies or social media platforms with different user characteristics [15].

Furthermore, online reaction behavior’s attributes are not mutually exclusive because a person can have multiple reactions after viewing a post, such as liking and/or sharing the post. Besides, we added an attribute called “nothing” to reflect that an individual may have no reaction at all, or a reaction that is not captured by the social media platform. For example, a user may laugh at a post but fail to “like” it after viewing. The “nothing” attribute is theoretically the same as “non-respondent bias” in survey research. Although there are other digital tracking tools to help infer viewers without any online reactions, researchers have been unable to directly access or retrieve such information since social media companies can decide what information can be available to researchers.

When investigating public intentions toward COVID-19 vaccination, acceptance can be theoretically associated with pro-vaccine individuals, rejection probably suggests those with anti-vaccine attitudes, and non-commitment might be regarded as a proxy for vaccine-hesitant people [10]. However, we acknowledge that there are limitations in this assumption, and need to be careful in how we interpret data and ascribe intentions based on our categorization of individuals. To better understand public discourse on social media, a promising ML technique—stance detection—can be applied to infer people’s attitudes toward a given topic [6]. For example, whether people support or oppose the COVID-19 vaccination. In addition to stance detection [6], a common way to infer attitudes in existing infodemic studies involves topic modeling and sentiment analyses [2]. Depending on models of sentiment analyses, emotions can be categorized at basic levels (i.e., positive, neutral, and negative) or more detailed levels, such as sadness, anger, happiness, joy [2]. However, according to our research experiences and other infodemic studies, sentiment analysis can still result in misclassifications regardless of levels [2]. Therefore, the proposed conceptual framework remains conventional to maintain emotions at basic levels with an additional level called “mixed” sentiment. The “mixed” attribute is added to address possible misclassifications in the “neutral” category resulting from sentiment analysis. When a tweet is categorized as “neutral,” this does not mean the tweet is “neutral” because it can actually be “positive,” “negative,” or “mixed” overall when interpreted, depending on its context [2]. Misclassifications often occur in ironic or humorous tweets [2]. The “mixed” feeling in the proposed conceptual framework refers to an equal amount of positive and negative feelings expressed simultaneously in a tweet without being “positive” or “negative” overall. For instance, if someone tweets an equal number of concerns and favors toward COVID-19 vaccines without explicit conclusions, this tweet can be regarded as “mixed” by humans, but is likely classified as “neutral” by sentiment analysis. However, we acknowledge that existing sentiment analysis techniques have not been sophisticated or advanced enough to recognize such “mixed” feelings. In addition, even humans cannot interpret mixed feelings consistently given external social-cultural factors, similar to humor differing between cultures. Therefore, improvements are still needed.

Our study has some limitations. Overall, more evaluations are needed since this is a new conceptual framework. Furthermore, given that the SoMeIL framework primarily focuses on social media, it is acknowledged that this proposed framework can only be useful in more digitalized populations, cultures, or nations. Besides, with new social media platforms being created, data formats and types can change given different platform designs. Therefore, the SoMeIL framework may need to be revised to reflect and investigate non-textual data, such as videos and images. Although there are advanced NLP and ML techniques that can analyze videos and images, these have not been well adapted in current infodemic social listening studies. Lastly, each social media platform has different user characteristics, rendering the data biased. Researchers will need to be careful when interpreting findings from different social media platforms even with the proposed SoMeIL conceptual framework.

As social media have integrated into people’s daily lives worldwide, its dominance will make health infodemics have greater impacts on people. Therefore, it is crucial to “listen to” public discourse on different social media platforms and address emerging confusions, questions, and even misinformation in a timely manner. Overall, the proposed SoMeIL conceptual framework has provided a preliminary yet quantifiable way for social listening. It is recommended that future pandemic preparedness recognizes the significant roles that social media plays in shaping public perception, disseminating information, and influencing behaviors during a health crisis. Incorporating social media into pandemic preparedness strategies besides others can enhance communication, information sharing, and response efforts.

### Conclusion

Although existing health behaviors, communications, and latest infodemic theories have been used in infodemic studies, these theories do not reflect the distinctive nature of social media in the current complex information ecosystems. Therefore, the SoMeIL conceptual framework is proposed to help future infodemic research. We acknowledge that the SoMeIL conceptual framework still needs validation for its efficacy, safety, and usability, and we anticipate that the framework will be revised as more studies are conducted in the future. However, the framework may help researchers to better understand public discourse and better infer collective behavioral intentions or behaviors. This may also help researchers to investigate how social media algorithms play an important role in being fed and actively feeding information to social media users given their online reaction behaviors.

## Data Availability

Theories and articles reviewed in this article can be found on PubMed, Scopus, PsycINFO, and Google Scholar.
