# Supplementary material for: Proposing a Conceptual Framework: Social Media Infodemic Listening for Public Health Behaviors
Source: Int J Public Health. 2024 Nov 14;69:1607394. doi: 10.3389/ijph.2024.1607394 (PMC11602303; doi:10.3389/ijph.2024.1607394)
Supplement: Supplementary file 1 [file DataSheet1.docx]

**Table S1.** Codebook used to analyze constructs for included theories (Proposing A Conceptual Framework: Social Media Infodemic Listening for Public Health Behaviors, Waterloo, Canada, 2024).

| Code | Definition | Example | Potential ML/NLP techniques |
| --- | --- | --- | --- |
| Health information | A post states any health-related information | Cue to action (Health Belief Model) | Topic modeling |
| Inferred attitude or perception | A user’s attitude towards a health information | Perceived susceptibility, perceived severity, perceived benefits, perceived barriers (Health Belief Model) | Topic modeling, stance detection |
| Inferred emotion | A user’s emotion towards a health information | Affective response (Risk Information Seeking and Processing Model) | Sentiment analysis |
| Inferred intention | A user’s behavioral intention to do something explicitly indicating in a post or suggested from inferred attitude and/or inferred emotion | Behavioral intention (Theory of Planned Behavior) | Topic modeling, stance detection, sentiment analysis |
| Behavior -- online | Reactions that are captured by a social media platform given its platform design | Behavior (Theory of Planned Behavior) | N/A |
| Behavior -- offline | Reactions that take place offline shared by users’ posts | Behavior (Theory of Planned Behavior) | Topic modeling, stance detection |

Table S2. Theories and models used in health infodemic research in the context of the COVID-19 pandemic (Proposing A Conceptual Framework: Social Media Infodemic Listening for Public Health Behaviors, Waterloo, Canada, 2024).

| **Theory/Model** | **Focus** | **Constructs** |
| --- | --- | --- |
| Behavioral and Social Drivers | Behavior | Confidence, Motivation, and Behavior |
| Capability, Opportunity, and Motivation lead to Behavior | Behavior | Capability, Opportunity, Motivation, and Behavior |
| Elaboration Likelihood Model | Attitude or Behavior | Motivation, Ability, and Opportunity to decide Central route or Peripheral route |
| Extended Parallel Process Model | Behavior | Threat and Efficacy |
| Health Belief Model | Behavior | Perceived susceptibility, Perceived severity, Perceived benefits, Perceived barriers, Modifying variables, Cues to action, and Self-efficacy |
| Risk Information Seeking and Processing Model | Attitude or Behavior | Combine both theory of planned behavior and heuristic systematic model |
| Social Cognitive Theory | Behavior | Behavioral capability, Observational Learning, Reinforcements, Expectations, Self-efficacy, and Reciprocal Determinism |
| Social Judgment Theory | Attitude | Latitude of Acceptance, Latitude of Non-commitment, and Latitude of Rejection |
| The Hype Loop | Behavior | Consume, Act, Sense, and Suggest |
| Theory of Planned Behavior | Behavior | Attitudes, Subjective norm, Perceived behavioral control, Behavioral intention, and Behavior |
| Theory of Reasoned Action | Behavior | Attitudes, Subjective norm, Behavioral intention, and Behavior |
| Transtheoretical Model | Behavior | Precontemplation, Contemplation, Preparation, Action, Maintenance, and Termination |
| Uses and Gratifications Theory | Behavior | Cognitive need, Affective need, Personal integrative need, Social integrative, and Tension release need |
